# Pathological Evolution and Internal Medicine Management of Nonalcoholic Fatty Liver Disease (NAFLD) in the Era of Metabolic Dysfunction-Associated Steatotic Liver Disease (MASLD)

**DOI:** 10.7759/cureus.86963

**Published:** 2025-06-29

**Authors:** Muhammad Moseeb Ali Hashim, Muhammad Aizaz Mohsin Khan, Muhammad Usama Ashraf, Saniya Mohsin, Kamran Zahoor, Javeria Niazi, Aiza Khan, Sania Muzaffar, Madiha Makhdumi, Omar Ahmed Ibad, Talha Kamran Khan, Sohaib Khalid

**Affiliations:** 1 Pathology and Laboratory Medicine, University of Missouri-Columbia, Columbia, USA; 2 School of Medicine, The University of Buckingham, Buckingham, GBR; 3 Internal Medicine, Changsha Medical University, Changsha, CHN; 4 Internal Medicine, Baqai Medical University, Karachi, PAK; 5 Pathology, Allama Iqbal Medical College, Lahore, PAK; 6 Pathology and Laboratory Medicine, Dow University of Health Sciences, Karachi, PAK; 7 Pathology and Laboratory Medicine, Baylor Univeraity Medical Center, Dallas, USA; 8 Pathology and Laboratory Medicine, Shifa College of Medicine, Islamabad, PAK; 9 Pathology and Laboratory Medicine, University of California Davis Health System, Sacramento, USA; 10 Histopathology, Chughtai Institute of Pathology, Lahore, PAK

**Keywords:** enhanced liver fibrosis (elf) test, fibrosis-4 (fib-4) score, internal medicine management of masld, liver fibrosis and steatohepatitis, magnetic resonance imaging-proton density fat fraction, metabolic dysfunction-associated steatohepatitis (mash), metabolic dysfunction-associated steatotic liver disease (masld), nonalcoholic fatty liver disease (nafld), non-invasive diagnostic tools in liver disease, vibration-controlled transient elastography (vcte)

## Abstract

Metabolic dysfunction-associated steatotic liver disease (MASLD), previously known as nonalcoholic fatty liver disease (NAFLD), is now recognized as the most prevalent chronic liver disease worldwide, driven by the rise in obesity, type 2 diabetes, and metabolic syndrome. The evolving nomenclature and understanding of MASLD necessitate updated insights into its pathophysiology, diagnostics, and internal medicine management.

A comprehensive literature review was conducted using PubMed, Scopus, Web of Science, and Cochrane databases for articles published between 2018 and 2025. Eligible studies included human-based clinical or translational research addressing MASLD pathogenesis, diagnostics, and management. Non-invasive scoring systems, pharmacotherapies, and multidisciplinary management strategies were evaluated.

The pathological progression of MASLD spans from simple steatosis to steatohepatitis, fibrosis, cirrhosis, and hepatocellular carcinoma. Key pathogenic mechanisms involve insulin resistance, adipokine imbalance, gut-liver axis dysregulation, and genetic polymorphisms such as PNPLA3. Diagnostic approaches have shifted toward non-invasive tools, including Fibrosis-4 (FIB-4), NAFLD Fibrosis Score, Enhanced Liver Fibrosis (ELF) score, Vibration-Controlled Transient Elastography (VCTE), and magnetic resonance imaging-proton density fat fraction (MRI-PDFF). Lifestyle modification remains the cornerstone of management, but promising pharmacologic therapies, such as glucagon-like peptide-1 (GLP-1) receptor agonists, vitamin E, pioglitazone, and resmetirom, are emerging. Multidisciplinary risk factor control, including diabetes, lipid, and blood pressure management, is critical. Emerging biomarkers and multi-omics technologies, alongside artificial intelligence, are redefining MASLD stratification and therapeutic monitoring.

Early identification and comprehensive management of MASLD are essential to prevent advanced liver disease and associated comorbidities. With evolving nomenclature, non-invasive diagnostics, and emerging therapies, internal medicine practitioners must adopt an integrative, multidisciplinary approach to care. Future research should prioritize personalized treatment strategies and health system integration to address the growing MASLD burden.

## Introduction and background

Nonalcoholic fatty liver disease (NAFLD) has become the most common chronic liver disorder globally, affecting nearly 25-30% of the population, with increasing prevalence driven by sedentary lifestyles, poor dietary habits, and rising obesity and diabetes rates [1]. NAFLD represents a spectrum of liver pathologies, ranging from benign hepatic steatosis to nonalcoholic steatohepatitis (NASH), which can progress to fibrosis, cirrhosis, and hepatocellular carcinoma (HCC) [1-3].

The disease is not confined to hepatic outcomes. Multiple studies have demonstrated its strong associations with cardiovascular disease, chronic kidney disease (CKD), atrial fibrillation, and extrahepatic malignancies [4-7]. As our understanding of its pathogenesis evolved, it became clear that the label "nonalcoholic" inadequately reflected the metabolic origins and heterogeneity of the disease. In 2023, international experts introduced a new term, metabolic dysfunction-associated steatotic liver disease (MASLD), to better encapsulate the metabolic root of the disease and establish a positive, rather than exclusionary, diagnostic criterion [8,9].

MASLD criteria now encompass patients with steatosis in the presence of at least one metabolic risk factor, such as type 2 diabetes mellitus (T2DM), obesity, or dyslipidemia, irrespective of alcohol intake. This has expanded the diagnostic reach and clinical relevance of the condition, allowing for more inclusive studies and improved patient stratification [10-12]. These changes bear significant implications for epidemiological surveillance, treatment planning, and clinical trial design.

This review presents a comprehensive analysis of the pathological evolution of NAFLD/MASLD and summarizes internal medicine management strategies. It aims to guide clinicians and researchers through updated nomenclature, diagnostic tools, and therapeutic interventions aligned with the current definition of MASLD.

## Review

Methodology and article selection

A structured literature search was conducted using four databases: PubMed (n=134), Scopus (n=92), Web of Science (n=58), and the Cochrane Library (n=44), covering studies published between January 2018 and March 2025. The search strategy used combinations of keywords including: “NAFLD,” “nonalcoholic fatty liver disease,” “MASLD,” “metabolic dysfunction-associated steatotic liver disease,” “NASH,” “fibrosis,” “diagnostic markers,” “non-invasive tools,” “biomarkers,” “pharmacotherapy,” “cardiometabolic comorbidities,” and “public health implications.”

Studies were eligible for inclusion if they were peer-reviewed, published in English, involved human subjects, and focused on the pathophysiology, diagnosis, treatment, or public health aspects of NAFLD, MASLD, or MAFLD. Eligible study designs included randomized controlled trials, cohort studies, cross-sectional analyses, systematic reviews, meta-analyses, clinical guidelines, and expert consensus statements.

After removing duplicate records (n=58), a total of 270 unique articles were screened by two independent reviewers. Of these, 76 articles were excluded due to lack of relevance to NAFLD/MASLD, 34 were preclinical or animal-only studies, 25 were editorials or narrative commentaries lacking original data, and 31 were excluded due to methodological limitations or unavailable full texts. Following full-text review and consensus resolution, 98 studies were included in the final synthesis of this review.

Data from selected articles were extracted into structured domains, including study type, population, diagnostic criteria, pathophysiological insights, clinical outcomes, and therapeutic or public health implications. Reference management was performed using Zotero (Corporation for Digital Scholarship, Fairfax, VA, USA), and citations were numbered in the order of appearance throughout the manuscript.

Pathological evolution of NAFLD/MASLD

**Figure 1:**
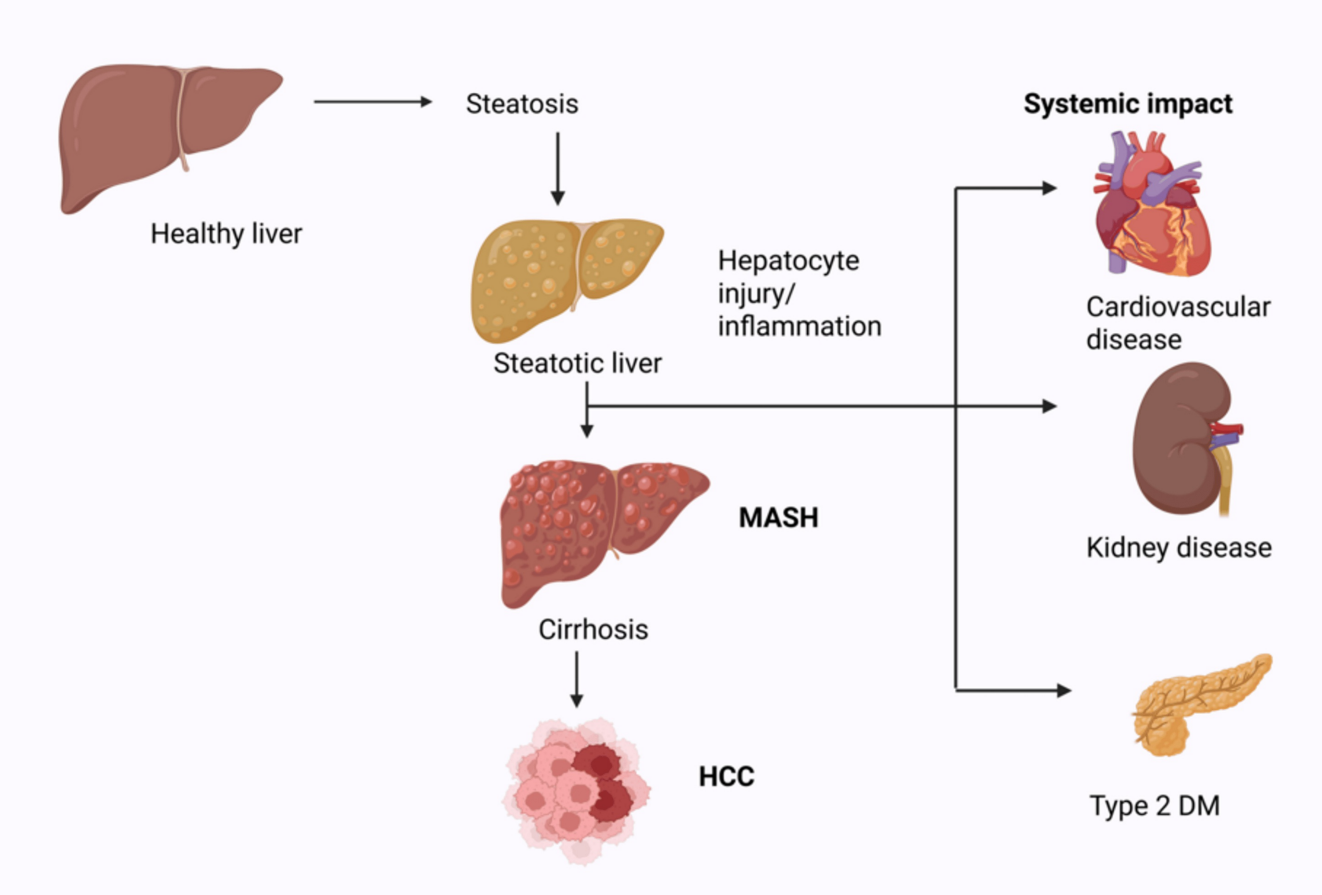
Pathophysiological progression of metabolic dysfunction-associated steatohepatitis (MASH) from hepatic steatosis to hepatocellular carcinoma (HCC), highlighting key histological stages and associated systemic complications, including cardiovascular disease, chronic kidney disease, and type 2 diabetes mellitus (T2DM). Credit: The image was created by the authors using BioRender.com.

The pathological progression of NAFLD, recently redefined as MASLD, represents a dynamic and multifactorial continuum driven by metabolic stress, systemic inflammation, genetic susceptibility, and environmental influences [13]. The condition initiates with hepatic steatosis, defined as the accumulation of triglycerides in more than 5% of hepatocytes, without secondary causes such as alcohol, medications, or viral infections [14]. While simple steatosis may remain clinically silent in many, a subset of patients progresses to metabolic dysfunction-associated steatohepatitis (MASH), which is histologically characterized by lobular inflammation, hepatocyte ballooning, and varying degrees of fibrosis [1,15]. Figure 1 illustrates the progressive stages of MASLD.

Histological Spectrum and Fibrogenesis

The histopathologic spectrum of MASLD includes four progressive stages: (1) simple steatosis, (2) steatohepatitis (MASH), (3) hepatic fibrosis, and (4) cirrhosis [16,17]. The transition to MASH is driven by lipotoxicity, oxidative stress, mitochondrial dysfunction, and activation of inflammatory signaling pathways [18]. Ballooning degeneration and Mallory-Denk body formation signal irreversible hepatocellular injury, which subsequently activates hepatic stellate cells and extracellular matrix deposition, leading to fibrosis [19]. Among all histological features, fibrosis stage has emerged as the most powerful predictor of liver-related events and all-cause mortality [20,21].

Recent longitudinal biopsy studies reveal that nearly 30-40% of patients exhibit fibrosis progression within five to seven years, even in the absence of baseline NASH [3]. Conversely, histological regression is possible with weight reduction, metabolic control, or targeted therapy, highlighting the importance of early intervention [22].

Immunopathogenesis and Inflammatory Crosstalk

The immune-driven pathogenesis of MASLD involves complex interactions between hepatocytes, Kupffer cells, monocyte-derived macrophages, neutrophils, and T cells [13,23]. Pattern recognition receptors (PRRs) such as toll-like receptors (TLRs) respond to gut-derived endotoxins and endogenous damage-associated molecular patterns (DAMPs), leading to activation of nuclear factor kappa B (NF-κB) and inflammasome pathways [24]. Kupffer cell polarization and immune cell recruitment further perpetuate chronic inflammation and fibrogenesis.

Adipokine dysregulation, characterized by elevated leptin, resistin, and tumor necrosis factor-alpha (TNF-α) levels and reduced adiponectin, exacerbates hepatic insulin resistance and contributes to cellular stress [25,26]. Single-cell transcriptomics and spatial mapping have revealed unique macrophage and lymphocyte subpopulations in MASH with pro-fibrotic gene expression signatures [27].

Gut-Liver Axis and Microbiota Interactions

The gut-liver axis plays a pivotal role in MASLD progression through dysbiosis, increased intestinal permeability, and altered bile acid signaling [28]. Translocation of bacterial endotoxins promotes hepatic inflammation and stellate cell activation. Metabolomics data reveal that short-chain fatty acids, secondary bile acids, and choline-derived metabolites may modulate immune responses and lipid accumulation in hepatocytes [29]. Probiotic and prebiotic interventions have shown modest benefit in modulating liver enzymes and inflammatory cytokines [30].

Genetic and Epigenetic Drivers of Progression

Several genetic polymorphisms modulate MASLD susceptibility and progression. The patatin-like phospholipase domain-containing protein 3 (PNPLA3) rs738409 variant (I148M) is the most robustly associated gene, conferring increased hepatic fat content and fibrosis [13]. Other risk alleles include transmembrane 6 superfamily member 2 (TM6SF2), membrane-bound O-acyltransferase domain-containing 7 (MBOAT7), hydroxysteroid 17-beta dehydrogenase 13 (HSD17B13), and glucokinase regulatory protein (GCKR) [31]. Interestingly, HSD17B13 variants may be protective in some subpopulations, emphasizing the need for ancestry-specific genetic analysis [32]. Key genetic variants associated with MASLD susceptibility and disease progression are summarized in Table 1.

**Table 1:** Genetic markers associated with MASLD This table presents key genetic variants linked to MASLD susceptibility, fibrosis progression, and interindividual variability in disease expression. PNPLA3: patatin-like phospholipase domain-containing protein 3; TM6SF2: transmembrane 6 superfamily member 2; MBOAT7: membrane-bound O-acyltransferase domain-containing 7; HSD17B13: hydroxysteroid 17-beta dehydrogenase 13; GCKR: glucokinase regulatory protein; MASLD: metabolic dysfunction-associated steatotic liver disease

Genetic variant	Clinical relevance
PNPLA3 (rs738409, I148M)	Strongest association with hepatic fat accumulation and fibrosis risk
TM6SF2	Promotes hepatic fat retention and fibrosis progression
MBOAT7	Linked to increased susceptibility to hepatic inflammation
HSD17B13	Protective in certain populations; reduces liver injury
GCKR	Associated with altered glucose and lipid metabolism

In parallel, epigenetic mechanisms, especially microRNAs (miRNAs) such as miR-122, miR-34a, and miR-192, modulate lipid metabolism, fibrogenesis, and insulin signaling [27]. Circulating miRNAs are being explored as non-invasive biomarkers for MASLD staging and therapeutic monitoring [33].

Progression to Cirrhosis and HCC

As fibrosis advances to cirrhosis, patients become vulnerable to hepatic decompensation, variceal bleeding, and HCC [34]. Notably, MASLD-related HCC can arise in non-cirrhotic livers, implicating alternative pathways such as oxidative DNA damage, telomere shortening, and oncogenic signaling [35]. Studies estimate that 15-30% of MASLD-HCC cases occur without cirrhosis, posing significant challenges for surveillance and early detection [36].

Alarmingly, patients with MASLD often lack access to regular surveillance, leading to late-stage HCC diagnosis and inferior prognosis [37]. Emerging models incorporating genetic, clinical, and biomarker-based risk scores may help identify at-risk individuals before they reach advanced disease [38].

Non-invasive diagnostic tools

The shift from invasive liver biopsy to non-invasive diagnostic methods marks a major advancement in the clinical management of NAFLD/MASLD. As the disease continues to rise globally and within the United States, reliable, accessible, and reproducible non-invasive tools are essential for screening, risk stratification, and longitudinal monitoring.

Serum Biomarkers and Scoring Systems

A range of serum-based tests are widely used in clinical practice to estimate fibrosis stage and overall liver disease severity. The alanine aminotransferase (ALT) and aspartate aminotransferase (AST) levels are typically elevated in MASLD, but they lack sensitivity and specificity in isolation [14]. Therefore, composite scores incorporating age, liver enzymes, platelet count, and other variables have become central to MASLD evaluation.

**Figure 2:**
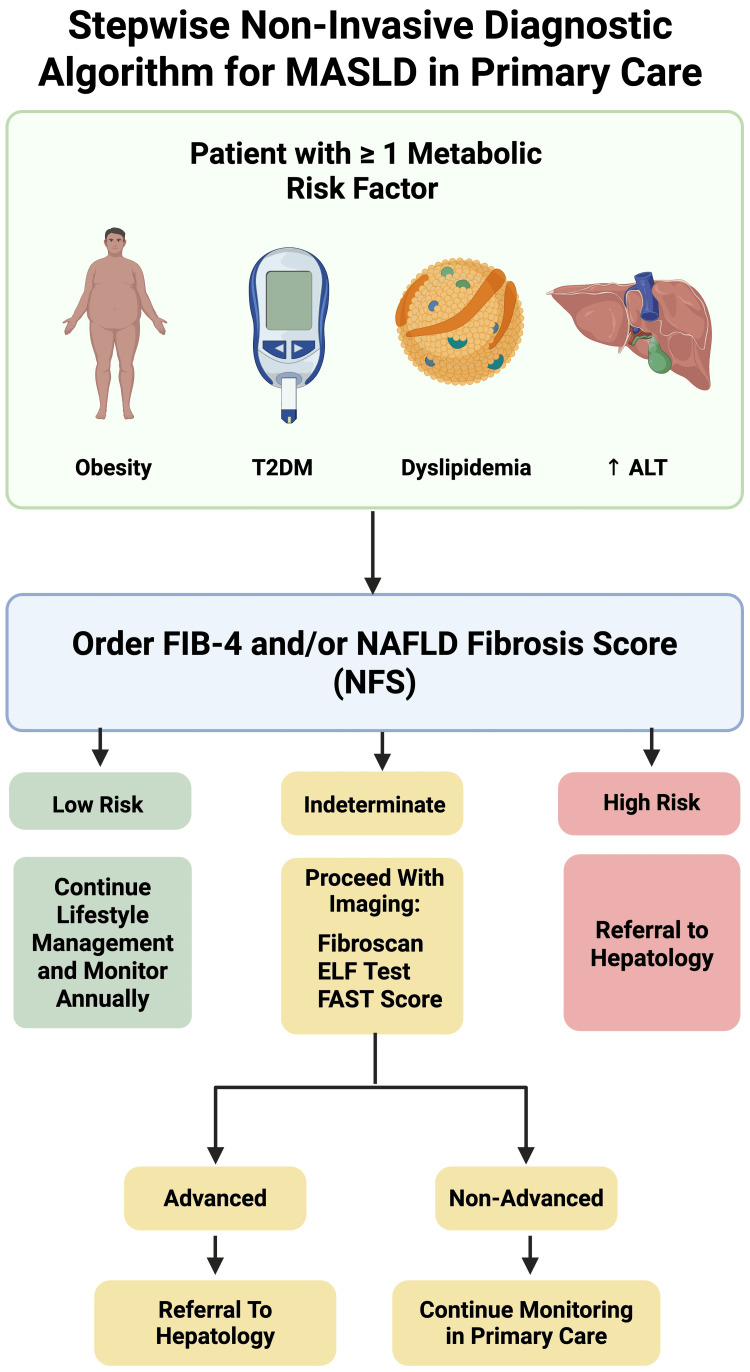
Stepwise non-invasive diagnostic algorithm for MASLD in primary care. Patients with metabolic risk factors undergo fibrosis-4 (FIB-4) and/or NAFLD fibrosis score (NFS) assessment to stratify fibrosis risk and guide further workup and referral. MASLD: metabolic dysfunction-associated steatotic liver disease; NAFLD: nonalcoholic fatty liver disease; ALT: alanine aminotransferase; ELF test: Enhanced Liver Fibrosis test; FAST score: FibroScan-AST score; T2DM: type 2 diabetes mellitus Credit: The image was created by the authors using BioRender.com.

The fibrosis-4 (FIB-4) index, which includes age, AST, ALT, and platelet count, is a simple, validated tool used to distinguish low-risk from high-risk fibrosis patients. It has been incorporated into many primary care screening protocols due to its affordability and accessibility [39]. Similarly, the NAFLD fibrosis score (NFS), which includes body mass index (BMI), age, diabetes status, AST/ALT ratio, platelet count, and albumin, provides reliable stratification of advanced fibrosis, particularly in community-based populations [21]. Figure 2 outlines a stepwise diagnostic algorithm for MASLD, illustrating how patients with metabolic risk factors can be efficiently stratified and referred based on fibrosis risk.

More advanced panels, such as the Enhanced Liver Fibrosis (ELF) test, combine multiple circulating fibrosis markers, including hyaluronic acid, tissue inhibitor of metalloproteinase 1 (TIMP-1), and procollagen III N-terminal peptide (PIIINP), demonstrating strong correlation with biopsy-proven fibrosis and predicting outcomes [40]. However, high cost and limited availability currently constrain its widespread use.

Imaging Modalities

Non-invasive imaging has become a cornerstone of MASLD assessment. Conventional abdominal ultrasound is typically the first-line imaging modality due to its widespread availability, low cost, and ease of use. It can detect moderate-to-severe hepatic steatosis but is less sensitive for mild fat infiltration (<20%) [41].

Vibration-Controlled Transient Elastography (VCTE), commercially known as FibroScan, measures liver stiffness as a surrogate for fibrosis and includes a controlled attenuation parameter (CAP) to estimate hepatic steatosis. It is non-invasive, point-of-care, and widely validated in MASLD populations [42]. A liver stiffness measurement (LSM) >12 kPa typically indicates advanced fibrosis or cirrhosis, while CAP >280 dB/m correlates with significant steatosis [43,44].

Magnetic resonance imaging-proton density fat fraction (MRI-PDFF) is the most accurate non-invasive modality for quantifying hepatic steatosis, correlating strongly with histologic fat content. MRI elastography (MRE) also offers high diagnostic accuracy for liver fibrosis but is limited by cost, availability, and the need for specialized equipment [45].

Indications for Liver Biopsy

Despite the rise of non-invasive tools, liver biopsy remains the gold standard for diagnosing NASH (MASH), staging fibrosis, and excluding other coexisting liver diseases [46]. However, its use is generally reserved for patients with diagnostic uncertainty, suspected advanced fibrosis, or for enrollment in clinical trials. Limitations include sampling variability, interobserver variability, procedural risks, and patient reluctance [47,48].

Clinical guidelines recommend considering biopsy in patients with indeterminate FIB-4 or NFS scores, discordant imaging and laboratory findings, or in those being evaluated for pharmacologic treatment [49]. In children and adolescents with suspected MASLD, biopsy remains an important tool to differentiate simple steatosis from more advanced pathology, although efforts are underway to validate pediatric non-invasive scores [50,51].

The evolving landscape of non-invasive diagnostics has revolutionized MASLD screening and monitoring. Incorporating these tools into routine internal medicine workflows enables early identification of at-risk patients and timely referral for hepatology care, reducing reliance on invasive procedures.

Internal medicine management strategies

Management of NAFLD/MASLD represents a multidisciplinary challenge that extends beyond hepatology and into the core of internal medicine. Given its strong associations with obesity, T2DM, dyslipidemia, hypertension, and cardiovascular disease, effective management requires an integrated, patient-centered approach that addresses both hepatic and extrahepatic risk factors [52].

Lifestyle Modifications: The Foundation of Therapy

Lifestyle modification remains the cornerstone of MASLD management. Weight loss through caloric restriction and physical activity has consistently shown benefit in reducing hepatic steatosis, inflammation, and fibrosis [53]. A sustained weight loss of ≥7% is associated with histological improvement in steatosis, while ≥10% weight reduction can lead to NASH resolution and even fibrosis regression [54]. However, less than 25% of patients achieve this level of weight loss in clinical practice, highlighting the need for structured lifestyle intervention programs [55].

Exercise, independent of weight loss, reduces hepatic fat and improves insulin sensitivity. Both aerobic and resistance training modalities are effective, with current guidelines recommending 150-300 minutes per week of moderate-intensity exercise [56]. Dietary patterns such as the Mediterranean diet, rich in monounsaturated fats, polyphenols, and fiber, have demonstrated favorable outcomes in reducing hepatic fat and improving metabolic parameters [57,58].

Pharmacologic Therapies Under Investigation and Use

Currently, there is no Food and Drug Administration (FDA)-approved pharmacologic treatment specifically for MASLD, but several agents have shown promise in targeting key pathways in steatohepatitis and fibrosis. Insulin sensitizers such as pioglitazone have demonstrated histological improvement in NASH, especially in patients with T2DM, but its use is limited by weight gain and concerns about long-term safety [59,60]. Vitamin E, an antioxidant administered at 800 IU/day, has shown benefit in non-diabetic patients with biopsy-proven NASH by reducing hepatocellular injury, but concerns about long-term cancer risk remain [61]. Glucagon-like peptide-1 (GLP-1) receptor agonists (GLP-1RAs) such as liraglutide and semaglutide promote weight loss and reduce hepatic steatosis and inflammation. Semaglutide in particular has shown robust histologic response in resolving NASH without worsening fibrosis in phase 2 trials [62,63]. Sodium-glucose co-transporter 2 (SGLT2) inhibitors not only improve glycemic control but also reduce liver fat and ALT levels, and are being explored for their role in MASLD management [64,65]. Farnesoid X receptor (FXR) agonists and thyroid hormone receptor-beta (THR-β) agonists, such as obeticholic acid (FXR agonist) and resmetirom (THR-β agonist), are under phase 3 evaluation for their antifibrotic effects. Resmetirom has shown significant reductions in liver fat content and NASH resolution in recent trials [66,67].

Bariatric Surgery

For patients with obesity and advanced fibrosis or NASH unresponsive to medical therapy, bariatric surgery offers a viable option. It leads to sustained weight loss, improved insulin sensitivity, and reversal of steatohepatitis in most patients [68]. Roux-en-Y gastric bypass and sleeve gastrectomy are the most studied procedures, with data supporting histological improvement in up to 85% of cases [69].

However, patient selection is crucial, and potential postoperative complications such as nutrient malabsorption and liver decompensation in cirrhotic patients must be considered [70,71].

Non-pharmacologic Adjuncts and Emerging Concepts

Modulation of the gut microbiota through probiotics, prebiotics, and fecal microbiota transplantation (FMT) is gaining attention as a novel therapeutic avenue [72,73]. While early-phase studies suggest favorable effects on liver enzymes and inflammatory markers, long-term safety and efficacy remain under investigation.

Additionally, recent studies have evaluated structured dietary interventions including low-carbohydrate, high-protein diets and intermittent fasting regimens, which have shown promise in reducing hepatic fat content and improving cardiometabolic parameters [74,75].

Emerging areas of interest include the use of omega-3 fatty acids, bile acid modulators, and combined metabolic pathway inhibitors that simultaneously target multiple components of the MASLD pathophysiology [76,77].

Multidisciplinary Risk Factor Management

Given the multisystemic nature of MASLD, effective management necessitates a coordinated, multidisciplinary approach that extends well beyond the liver. Internists and primary care providers are central to this effort, particularly in managing metabolic comorbidities that drive disease progression and increase cardiovascular risk. T2DM, hypertension, dyslipidemia, and CKD are frequently coexisting conditions that significantly impact patient outcomes. Glycemic control should be optimized using agents with hepatic benefits, particularly GLP-1 receptor agonists and SGLT2 inhibitors, which not only improve insulin sensitivity but also promote weight loss and reduce hepatic steatosis [78,79]. Dyslipidemia should be aggressively treated, with statins considered safe and effective for reducing cardiovascular events in MASLD patients despite previous concerns regarding hepatotoxicity [80].

Hypertension management, including renin-angiotensin-aldosterone system (RAAS) blockade, may offer additional benefits in attenuating hepatic fibrosis and protecting renal function. Regular surveillance for CKD, using estimated glomerular filtration rate (eGFR) and albuminuria measurements, is essential given the bidirectional relationship between MASLD and renal dysfunction [81]. In addition, cardiovascular risk stratification and management, including lifestyle counseling, antiplatelet therapy when indicated, and smoking cessation, are critical components of holistic care. Multidisciplinary collaboration between hepatologists, endocrinologists, cardiologists, nephrologists, dietitians, and primary care physicians ensures that both hepatic and systemic risk factors are addressed comprehensively and continuously throughout the disease course [82].

Emerging therapies and biomarkers

The treatment landscape of MASLD is evolving rapidly, driven by a deeper understanding of disease mechanisms and the identification of novel molecular targets. In parallel, emerging biomarkers, ranging from circulating proteins to genomic signatures, are revolutionizing disease detection, prognosis, and therapeutic monitoring.

Biomarkers for Disease Activity and Fibrosis

The development of non-invasive biomarkers has addressed critical limitations of liver biopsy. Among these, cytokeratin-18 (CK-18), a fragment released during hepatocyte apoptosis, is the most extensively studied serum biomarker for distinguishing NASH from simple steatosis. Elevated CK-18 levels correlate with histologic ballooning and inflammation and have shown moderate diagnostic accuracy when combined with other markers [83]. A summary of potentially promising biomarkers currently under investigation for MASLD diagnosis and monitoring is presented in Table 2.

**Table 2:** Emerging biomarkers for MASLD diagnosis and monitoring This table highlights circulating biomarkers and non-invasive scoring systems under investigation for diagnosing and monitoring disease progression in MASLD. CK-18: cytokeratin-18; ELF: Enhanced Liver Fibrosis; AST: aspartate aminotransferase; miR: microRNA; MASLD: metabolic dysfunction-associated steatotic liver disease; NASH: nonalcoholic steatohepatitis; FAST score: FibroScan-AST score

Biomarker	Clinical Utility
CK-18	Differentiates NASH from steatosis; apoptosis marker
ELF Score	Predicts advanced fibrosis and liver-related outcomes
FAST Score	Combines FibroScan and AST to detect active NASH
miR-122/miR-192	Circulating microRNAs for disease activity
Exosomal markers	Under investigation for diagnosis and monitoring

The ELF test, which measures hyaluronic acid, TIMP-1, and PIIINP, is validated as a fibrosis staging tool and has shown prognostic value in predicting liver-related events [84]. Likewise, the FibroScan-AST (FAST) score, combining transient elastography (TE) and AST, enables identification of patients with active MASH and significant fibrosis in a non-invasive manner [85].

Emerging candidates include exosomal markers, miRNAs, and metabolomic signatures that reflect lipid dysregulation and hepatocyte injury. For instance, miR-122 and miR-192 are under investigation as biomarkers of disease severity and treatment response [27]. Multi-analyte panels and proteomic classifiers are being incorporated into clinical trial endpoints to capture therapeutic efficacy more accurately than histology alone.

Novel Pharmacologic Agents and Clinical Trials

Therapeutic innovation in MASLD is focused on disrupting key fibrogenic, inflammatory, and metabolic pathways. One of the most promising classes includes fibroblast growth factor 21 (FGF21) analogs, such as pegozafermin and efruxifermin, which enhance lipid oxidation, reduce hepatic fat, and improve insulin sensitivity. Early-phase trials have demonstrated reductions in liver fat and improvements in liver enzymes and fibrosis markers [86].

THR-β agonists, such as resmetirom, have shown significant reductions in hepatic fat fraction and improvements in histological features of MASH in phase 3 trials, positioning them as potential first-line pharmacotherapies [13]. Other agents in development include pan-peroxisome proliferator-activated receptor (pan-PPAR) agonists (e.g., lanifibranor), C-C motif chemokine receptor types 2 and 5 (CCR2/5) inhibitors, acetyl-CoA carboxylase (ACC) inhibitors, and stearoyl-CoA desaturase-1 (SCD-1) inhibitors, each targeting distinct aspects of MASLD pathophysiology [87-90].

Combination therapies, such as glucagon-like peptide-1 receptor agonists (GLP-1RAs) with FXR or PPAR agonists, are under investigation to enhance efficacy by addressing multiple pathogenic axes simultaneously. The SYNERGY-NASH trial, for example, demonstrated that tirzepatide, a dual glucose-dependent insulinotropic polypeptide (GIP)/GLP-1 receptor agonist, led to the resolution of MASH and weight reduction in patients with T2DM [91].

Multi-omics and Artificial Intelligence (AI) in Risk Stratification

Advanced technologies such as multi-omics profiling (genomics, transcriptomics, proteomics, and metabolomics) are being integrated into MASLD research to identify novel disease subtypes and therapeutic targets. These approaches enable precise patient phenotyping and identification of drug responders versus non-responders [92].

AI and machine learning algorithms are also transforming MASLD management by improving diagnostic accuracy and predictive modeling. AI-powered analysis of imaging (e.g., MRI-PDFF, elastography) and clinical data can non-invasively assess fibrosis progression, predict treatment response, and optimize patient selection for clinical trials [93].

These innovations are moving MASLD care into an era of precision hepatology, where individualized risk stratification and therapy selection are possible based on molecular and digital data integration. The future of MASLD management will likely involve routine use of composite biomarkers and AI-assisted platforms for screening, monitoring, and treatment guidance.

Policy and practice implications

The recent redefinition of NAFLD to MASLD carries significant implications for clinical practice, research, and public health policy. This paradigm shift affects not only hepatologists but also internists, endocrinologists, pathologists, and primary care physicians, who are increasingly at the frontline of MASLD detection and management.

MASLD Redefinition: What Changes for Internists and Pathologists?

The transition to MASLD, introduced by expert consensus in 2023, replaces the exclusionary “nonalcoholic” label with a positive diagnostic criterion based on hepatic steatosis and the presence of at least one metabolic risk factor [94]. This shift removes the ambiguity associated with defining "nonalcoholic" intake and eliminates unnecessary stigma, thereby improving diagnostic clarity in primary care and internal medicine settings.

For internists, this redefinition mandates increased vigilance in patients with obesity, T2DM, or metabolic syndrome. Primary care workflows must adapt to incorporate liver risk stratification using tools like FIB-4 and NFS at routine metabolic visits [95]. For pathologists, the MASLD nomenclature requires updated histopathology reporting templates that align with the steatosis-activity-fibrosis (SAAF) and NAFLD activity score systems and clearly distinguish MASH from simple steatosis [96].

This shift also underscores the importance of biopsy-independent diagnosis, enabling broader application of non-invasive tools for staging and monitoring MASLD in non-specialist settings.

United States Preventive Services Task Force (USPSTF) Recommendations and Screening Gaps

Despite its rising burden, MASLD remains underdiagnosed and under-prioritized in U.S. preventive health guidelines. The USPSTF currently does not have routine screening guidelines for MASLD in asymptomatic adults due to insufficient evidence on long-term benefits, treatment efficacy, and cost-effectiveness [97].

However, growing data suggest that targeted screening in high-risk populations, such as individuals with diabetes, obesity, or elevated transaminases, can identify advanced fibrosis earlier, reducing long-term complications [98]. Professional societies, including the American Association for the Study of Liver Diseases (AASLD), American Diabetes Association (ADA), and European Association for the Study of the Liver (EASL), now endorse non-invasive fibrosis risk assessment in patients with metabolic risk factors, even in the absence of symptoms [78].

Advocacy is needed to update USPSTF guidance in light of recent evidence and therapeutic advancements, particularly as emerging pharmacologic agents become available. Expanding screening eligibility and insurance coverage for tools like FibroScan and ELF testing will be crucial for equitable access to MASLD care.

Integration Into Primary Care and Population Health Strategies

A population health approach to MASLD requires integrating liver assessment into routine chronic disease management, particularly in patients with diabetes, cardiovascular disease, and obesity. This includes embedding FIB-4 calculators into electronic medical records, providing clinical decision support for high-risk patients, creating referral pathways for hepatology evaluation, and training primary care physicians and nurse practitioners in MASLD recognition and staging.

Such integration has already shown success in health systems that implemented automated fibrosis score alerts and elastography-based liver clinics. Furthermore, public health campaigns to raise MASLD awareness among patients and clinicians are necessary, especially in underserved populations where liver disease is often missed until late stages.

On the policy level, incorporating MASLD into national liver disease surveillance programs and Centers for Medicare and Medicaid Services (CMS) quality metrics could promote accountability and data collection on outcomes. As MASLD emerges as a leading cause of cirrhosis and HCC, early detection through primary care integration will be critical to reversing disease burden trends.

## Conclusions

NAFLD, now redefined as MASLD, has emerged as a major global health burden, intimately linked to the metabolic syndrome and the rising prevalence of obesity, T2DM, and sedentary lifestyles. This review underscores the systemic nature of MASLD and its progression from hepatic steatosis to steatohepatitis, fibrosis, cirrhosis, and HCC. As the most prevalent chronic liver condition in the United States, MASLD demands early recognition and management within primary care and internal medicine frameworks. A combination of non-invasive diagnostic tools, including serum biomarkers and imaging modalities, now enables accurate staging and risk stratification without liver biopsy in most patients. These tools facilitate the identification of individuals at risk of progression, particularly those with advanced fibrosis, who benefit most from intensive intervention. While lifestyle modification remains the cornerstone of treatment, pharmacotherapy options, including GLP-1 receptor agonists, vitamin E, and investigational agents like resmetirom and FGF21 analogs, are rapidly evolving, offering hope for effective medical therapy in the near future. Internists and multidisciplinary care teams play a central role in cardiometabolic risk factor control, including management of diabetes, hypertension, and dyslipidemia. As MASLD is increasingly recognized as a multisystem disease, collaborative models that integrate hepatology, endocrinology, cardiology, and primary care are essential.

Looking ahead, multi-omics technologies, AI-enhanced risk modeling, and novel biomarkers will likely revolutionize personalized care and therapeutic monitoring in MASLD. Equally important is the need for policy-level change, including updated USPSTF screening guidelines, broader insurance coverage of non-invasive tools, and inclusion of MASLD metrics in national quality measures. In conclusion, early detection and proactive management of MASLD can prevent progression to end-stage liver disease, reduce cardiovascular mortality, and improve population health outcomes. Continued research, interdisciplinary collaboration, and policy advocacy are needed to address knowledge gaps and to translate scientific advances into clinical and public health impact.
